# Comparison between C4d immunohistochemical staining and other clinical-histopathological findings in IgA nephropathy

**DOI:** 10.37796/2211-8039.1118

**Published:** 2021-06-01

**Authors:** Tala pourlak, Seyyed Hamed Sharif Arani, Sima Abediazar, Hossein Samadi Kafil

**Affiliations:** a Kidney Research Center, Tabriz University of Medical Sciences, Tabriz, Iran; b Pharmaceutical Nanotechnology Research Center, Faculty of Medicine, Tabriz University of Medical Sciences, Tabriz, Iran; c Drug Applied Research Center, Tabriz University of Medical Sciences, Tabriz, Iran

**Keywords:** IgA nephropathy, C_4_d immunohistochemical staining

## Abstract

**Introduction:**

IgAN occurs following abnormal IgA deposition in the glomerular mesangial regions. It is the most common primary glomerular disease and one of the causes of ESRD, so it is necessary to identify clinical and histopathological findings that predict progression to ESRD. In the physiopathology of this disease, C_4_d causes serious renal injuries and should be counted as a significant prognostic factor too. This study examined C_4_d biomarker and compare it with findings affecting prognosis, to determine the predictive value of C_4_d in progression to ESRD in IgAN.

**Materials and methods:**

In this study, all biopsy samples of IgAN patients who referred to Imam Reza Hospital in Tabriz were collected for four years. Their samples were evaluated C_4_d immunohistochemical staining and positive samples have compared with Clinical-histopathological findings affecting prognosis.

**Results:**

In this study, C_4_d positivity showed a significant association with mesangial hypercellularity (p = 0.001), segmental glomerulosclerosis (p = 0.003), and endocapillary hypercellularity (p = 0.001); however, it did not show a significant relationship with tubular atrophy/interstitial fibrosis (p = 0.08). The study also found that C_4_d positivity was significantly (p < 0.05) correlated with hypertension, increased proteinuria, hematuria, high creatinine, and decreased mean eGFR.

**Conclusion:**

This study showed that immunohistochemical staining of C_4_d is a useful method for evaluating the prognosis of the severity of renal injuries in patients with IgAN and could be a valuable alternative for most Clinical-histopathological factors routinely used as predictive factors for its progression to ESRD, especially when the biopsy specimen size is small and insufficient for other studies.

## 1. Introduction

IgA nephropathy (IgAN) is one of the most common primary glomerular diseases worldwide [1]. The disease is a type of proliferative glomerulonephritis that occurs following abnormal IgA deposition in the mesangial region of the glomeruli. In the physiopathology of this disease, the complement system, especially the subtype C4d, plays an essential but unknown role by activating the lectin pathway [2]. On the other hand, it has been observed that in patients with activated lectin pathway, the risk of severe renal failure is increased and is significantly reduced within ten years [3]. Based on this evidence, it seems that C4d can be considered a significant prognostic factor as an indicator of lectin pathway activation. This disease is one of the most leading causes of end-stage renal disease (ESRD), so it is crucial to identify Clinical-histopathological factors that predict progression to ESRD. These factors include high blood pressure at the time of diagnosis [4–6], Any increase in urine protein concentrations especially values above 3.5 g in 24-hour urine collection at the onset or persistence of symptoms [4–12], persistent isolated hematuria at the time of symptoms [7, 8, 13, 14], high serum creatinine levels [7, 8, 15–18], and decreased Mean eGFR [7–10, 15, 19, 20] are among the factors that predict poor prognosis for renal survival in patients with IgAN. There are some critical histological factors in determining the prognosis of IgA nephropathy, including mesangial hypercellularity, endocapillary hypercellularity, segmental glomerulosclerosis, and tubular atrophy/interstitial fibrosis (abbreviated MEST), which is associated with adverse renal outcomes [21]. Renal biopsy is requisite for the diagnose of IgA nephropathy [22]; however, due to the invasive nature of this procedure, as well as its importance in determining prognosis in the choice of treatment intensity at the onset of the disease [23, 24] and the role of C4d subtype in the induction of severe renal failure, it would be necessary to unravel the role of C4d as a critical prognostic factor, especially in cases where the biopsy specimen size is small.

## 2. Materials and Methods

**Fig. 1:**
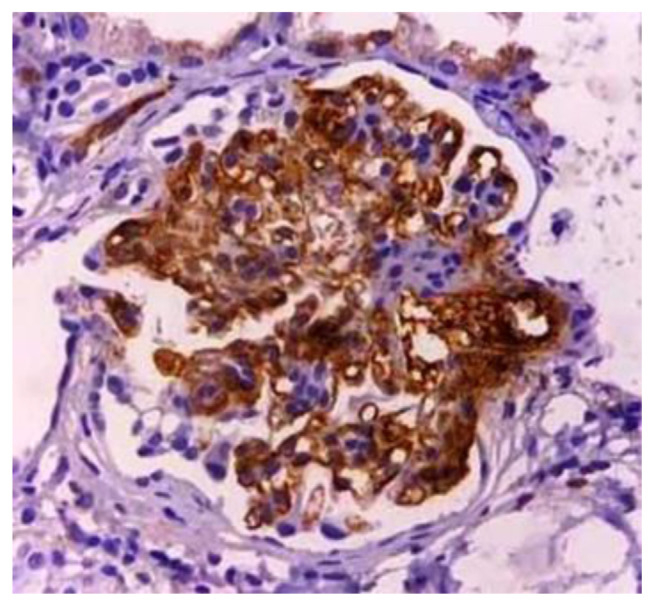
Positive immunohistochemical staining of C4d. Strong immunoreactivity and diffused C4d deposition are evident in the glomerular mesangial region (×400 magnification).

In this study, all patients with renal diseases who referred to Imam Reza Hospital in Tabriz were sent to the Pathology Department of this hospital between March 2015 and September 2019. The selection of the biopsy specimen was based on IgAN confirmation by immunofluorescence (IF) studies. The inclusion criteria of biopsy specimens were 1) The severity of IgA depositions ≥2+ [22] and negative C1q depositions in the IF studies [25]., 2) having at least six glomeruli that fixed in formalin 10% [22] and evaluated by hematoxylin and eosin (H&E) staining. For the analysis of the histological criteria according to the Oxford classification, nine serial sections from each specimen were providing and staining by H&E, Jones’ methenamine silver, Periodic acid–Schiff (PAS) [22]. The criteria included the presence of mesangial hypercellularity (more than 50% of glomeruli must have more than three mesangial cells as positive Specimens or cases) [22], segmental glomerulosclerosis (any amount of glomerular sclerosis as positive or cases) [22], endocapillary hyperplasia (any amount of hypercellularity in endocapillary areas as positive Specimens or cases) [22], and tubular atrophy/interstitial fibrosis (if present it must have more than 25% of the specimen to be included as positive or cases [22], on the other hand, another histological section was prepared and mounted on a charged slide for immunohistochemical staining with a rabbit anti-C4d monoclonal IgG antibody (BioCare Medical LLC). Specimens with strong and diffused anti-C4d antibody deposition in 75% of glomeruli in the mesangial region were known as positive cases (Fig. 1) [26]. Clinical and laboratory findings including high blood pressure (blood pressure 140/90 mm Hg and higher at the time of diagnosis) [4,26], proteinuria (excretion of over 400 mg of protein in 24-hour urine collection) [22]., persistent positive hematuria (with +1 dipstick strip or more during two consecutive urinalysis tests [7, 8, 13, 14], high creatinine (serum creatinine concentration above 1.4 mg/dL) [8] and eGFR^1^ reduction (60–90 ml/min per 1.73 m^2^ of body surface area as mild reduction, 30–60 ml/min per 1.73 m^2^ of body surface area as an average reduction, and less than 30 ml/min per 1.73 m^2^ of body surface area as severe reduction) [7–10, 15, 19, 20] that collected based on physical examination and laboratory testing at the beginning of referred.

### 2.1. Statistical analysis

Demographic, clinical, and laboratory data were evaluated retrospectively. Statistical analysis was carried out using SPSS software version 21.0. Based on the qualitative and quantitative nature of the test, the results were analyzed using Pearson or Spearman tests. Finally, the data were depicted in the form of Tables and Figures.

## 3. Results

**Fig. 2:**
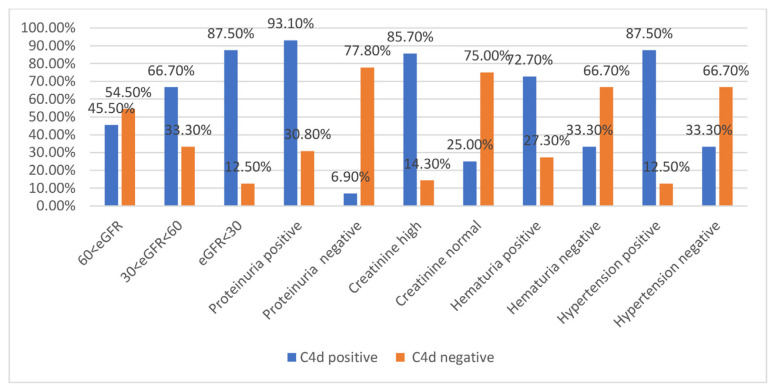
Comparison of Clinical-pathology factors with C4d immunoreactivity.

Out of a total of 51 patients with IgAN, only 48 met the required criteria to be included in our study. The age range of patients was between 15 and 63 years, and most of them were in the age range of 30 to 40 years, with a frequency of 33.3%. Among patients, 66.7% were male, and 33.3% were female. Among IgAN patients, 29 patients (60.4%) were immunohistochemically positive for C4d, while 19 patients (39.6%) were negative for this protein. In our study, there was a direct, strong, and significant correlation between the association of mesangial hypercellularity, endocapillary hypercellularity, and segmental glomerulosclerosis with C4d immunoreactivity in patients with IgAN (P < 0.05). It was also shown that most IgAN patients who were immunohistochemically positive for C4d were significantly more likely to have mesangial hypercellularity, endocapillary hypercellularity, and segmental glomerulosclerosis in renal histological specimens (P < 0.05) (Table 1). In our study, although most patients with IgAN who were positive for C4d had tubular atrophy/interstitial fibrosis in renal biopsy specimens, such a correlation was not statistically significant (P-value = 0.081) (Table 1). The statistical analysis showed that there was a significant association between decreased eGFR and increased immunoreactivity for C4d marker in patients with IgAN (P-value = 0.032), as the mean eGFR was 52.00 ml/min for patients who were positive for C4d, while it was 68.053 ml/min for patients who were negative for this marker (Table 2). Besides, a direct, strong, and significant correlation was detected between the association of high blood pressure with C4d immunoreactivity in patients with IgAN (P-value = 0.000) (Fig. 2). It has also been shown that most IgAN patients who were positive for C4d also had significantly higher values of (P-value = 0.012) (Table 5). Our findings demonstrated a direct, strong, and significant correlation between hematuria and C4d immunoreactivity in patients with IgAN (P-value = 0.01) (Fig. 2). It was also shown that most IgAN patients who were positive for C4d in immunohistochemical evaluations were significantly more likely to have hematuria (P-value = 0.0005) (Table 5). Also, a direct, strong, and significant association was found between high creatinine levels and C4d immunoreactivity in patients with IgAN (P-value = 0.000) (Table 3). It was also indicated that most IgAN patients who were positive for C4d in immunohistochemical assessments also had significantly higher concentrations of creatinine (P-value = 0.005) (Table 5). On the other hand, the results showed that IgAN patients who were positive for C4d in immunohistochemical evaluations had a higher average creatinine level compared with patients who were negative for this marker (2.05 mg/dl vs. 1.22 mg/dl) (Table 3). The results showed a direct, strong, and significant relationship between proteinuria with C4d immunoreactivity in patients with IgAN (P-value = 0.000) (Table 4). It was also shown that most IgAN patients who were positive for C4d also had a significant extent of proteinuria (P-value = 0.000) (Table 5). Our study indicated that IgAN patients who were positive for C4d also had a higher average protein excretion in their urine compared with C4d-negative patients (2.24 g/day vs. 1.09 g/day) (Table 4).

## 4. Discussion

IgA nephropathy is a common kidney disease that, through IgA deposition and with various mechanisms such as C4d deposition in the kidney leads to changes in clinical and histopathologic signs for the patient, and play an important role in the future for prediction of prognosis of the disease. In this investigation, we tried to study the predictive value of C4d as a new marker in association with other criteria affecting prognosis.

C4d plays a significant role in activating the lectin pathway [2], and by producing a variety of pro-inflammatory mediators, it causes mesangial hypercellularity [27, 28] as well as focal And or segmental glomerulosclerosis [3]. On the other hand, sub-endothelial C4d deposits [22], especially in the glomerular area [29], can lead to damage to podocytes through endocapillary proliferation [22]. As a result, the association between C4d and histological factors affecting prognosis in our study and other studies [26,30–32] suggests a negative effect of C4d on prognosis. However, the association between C4d positivity and tubular atrophy/interstitial fibrosis in the present research, unlike other studies [26,30–32], could be due to the higher specificity of the monoclonal antibody used in our study. However, higher positive C4d cases in the study group without tubular atrophy/interstitial fibrosis may be because of more important than C4d deposition in glomerular damage than extra-glomerular injury [29], as well as concomitant glomerular damage with tubular atrophy and interstitial fibrosis in positive C4d cases. Glomerular C4d deposition, followed by renal failure [34], impairs glomerular filtration, and increases serum creatinine as two factors affecting prognosis. Our study, similar to other studies [26,31,33], showed that C4d positivity with a decrease in mean eGFR to less than 60 ml/min caused significant destruction in renal function. Our study, similar to other studies, showed that C4d positivity with a decrease in mean eGFR to less than 60 ml/min caused significant destruction in renal function. On the other hand, according to our study and other previous researches [32] [26], C4d-negative patients had an average creatinine excretion of less than 1.25 mg/dl, while C4d-positive patients had a moderate creatinine level above 1.68 mg/dl, which according to previous studies can respectively lead to ESRD by 2.5% and 71%, within ten years [8]. The co-occurrence between C4d positivity and high blood pressure in our study and other investigations [26,31,33] can be exhibited severity renal injury in the kidney of IgAN patients. C4d deposits in the mesangial area can probably be through the proliferation of mesangial cells, secretion of extracellular matrix components [35]. Previous studies showed C4dstaining was more correlative with the endocapillary proliferation score of the Oxford classification, than with the mesangial changes [36]. Increased expression of βTGF, and activation of the renin-angiotensin system [37–39] lead to hypertension, renal injury and finally poor prognosis in the IgAN patients. The present study confirmed the concurrence of C4d deposition and hematuria. The emergence of hematuria at the time of the beginning of disease may be correlated with a higher risk of ESRD in the future [7, 8, 13, 14]. Also, the presence of RBCs inside the tubules, as a result of hematuria, causes severe injuries to the kidneys by causing acute tubular necrosis [7, 8, 13, 14]. Therefore, regardless of the damage mechanism of hematuria, a positive C4d will adversely influence the patient’s prognosis. Our study, similar to other studies [26,31,33], showed that C4d negativity causes average proteinuria of about 1 gram per day, whereas C4d positivity leads to an increase in proteinuria of more than 1 gram per day. Such an effect stems from C4d glomerular deposition and the resulting glomerular damage [34]. Therefore, due to the negative effect of proteinuria increase on prognosis, a positive C4d will result in a poor prognosis for the patient.

Finally, it can be inferred that C4d is a useful biomarker in assessing the prognosis of the severity of renal injury in IgAN patients and could be a worthy alternative to most clinical-histopathological factors predicting the progression towards ESRD.

## Figures and Tables

**Table 1:** Relationship between histopathologic factors and C4d immunoreactivity.

Histological	Category	C_4_d		P-Value	Chi-Square
				
		Positive	Negative		
Mesangial hypercellularity	Positive	23 (82.1%)	5 (17.9%)	0.000	13.264
	Negative	6 (30%)	14 (70%)		
Endocapillary proliferation	Positive	20 (87%)	3 (13%)	0.000	13.006
	Negative	9 (36%)	16 (64%)		
Segmental glomerulosclerosis	Positive	23 (79.3%)	6 (20.7%)	0.001	10.936
	Negative	6 (31.6%)	13 (68.4%)		
The proportional of tubular/atrophy and interstitial fibrosis	Positive	15 (75%)	5 (25%)	0.081	3.049
	Negative	14 (50%)	14 (50%)		

**Table 2:** The association between eGFR and C4d immunoreactivity.

		eGFR			Spearman Correlation		Mean ± S D
				
		>60	30–60	30>	P-Value	Phi	
C4d	Positive	10 (45.5%)	12 (66.7%)	7 (87.5%)	0.032	−0.311	52.00 ± 25.53
	Negative	12 (54.5%)	5 (33.3%)	1 (12.5%)			68.053 ± 22.9

**Table 3:** The relationship between creatinine and C4d immunoreactivity.

		Creatinine		Pearson Correlation			Mean ± SD
				
		Creatinine positive	Creatinine negative	Chi-Square	P-Value	Phi	
C4d	Positive	24 (85.7%)	5 (25%)	17.938	0.000	0.612	2.05 ± 1.15
C4d	Negative	4 (14.3%)	15 (75%)				1.22 ± 0.37

**Table 4:** The correlation between proteinuria and C4d immunoreactivity.

		Proteinuria		Correlation test results		Mean ± SD
				
		Proteinuria positive	Proteinuria negative	P-Value	Phi	
C4d	Positive	27 (93.1%)	2 (6.9%)	0.000	0.517	2.24 ± 1.32
	Negative	12 (30.8%)	7 (77.8%)			1.09 ± 1.25

**Table 5:** Relationship between Clinic-histopathologic factors in patients with positive C4d based on the binomial distribution.

		Frequency C_4_d positive	Observed Proportion	P-Value
Mesangial hypercellularity	Positive	23	79%	0.001
	Negative	6	21%	
Endocapillary proliferation	Positive	20	69%	0.003
	Negative	9	31%	
Segmental glomerulosclerosis	Positive	23	79%	0.001
	Negative	6	21%	
Hypertension	Positive	21	72%	0.012
	Negative	8	28%	
Hematuria	Positive	24	83%	0.0005
	Negative	5	17%	
Creatinine	Positive	24	83%	0.005
	Negative	5	17%	
Proteinuria	Positive	27	93%	0.000
	Negative	2	7%	

## Footnotes

1 eGFR (ml/min per 1.73 m^2^)=(186 x serum creatinine-1.154) x (age-0.203) x (0.742 if female) x (1.21 if black).
